# Supplementary material for: Cortistatin neurons in the prelimbic cortex regulate seizure susceptibility in female mice via BDNF–TrkB signaling
Source: bioRxiv. 2026 Feb 1:2026.01.28.702318. Preprint. [Version 1] doi: 10.64898/2026.01.28.702318 (PMC12873827; doi:10.64898/2026.01.28.702318)
Supplement: 1 [file NIHPP2026.01.28.702318V1-supplement-1.pdf]

bioRxiv preprint doi: https://doi.org/10.64898/2026.01.28.702318; this version posted February 1, 2026. The copyright holder for this preprint (which was not certified by peer review) is the author/funder, who has granted bioRxiv a license to display the preprint in perpetuity. It is made available under aCC-BY 4.0 International license.

8 | Supplementary Figures

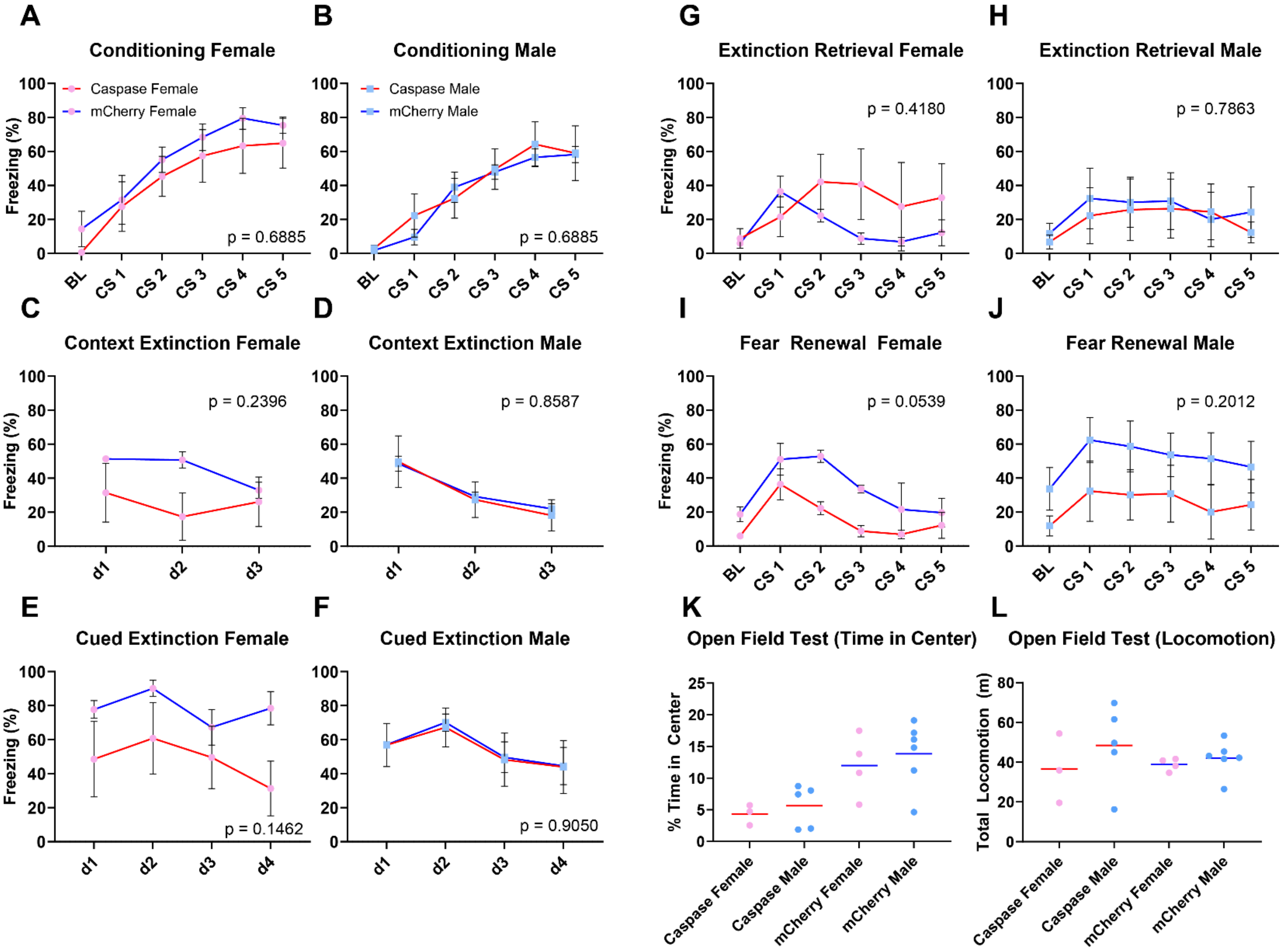

Fig. S1. Cst+ neuron ablation shows no sex differences during fear conditioning. A-L) When broken down by sex, there were no observed differences in any phase of the fear conditioning paradigm including during conditioning (A-B), context extinction (C-D), Cued Extinction (E-F), fear extinction retrieval (G-H),or fear renewal (I-J). (K-L) There were no observed differences between groups or sex in open field in time spent in center (K) or total locomotion (L).

bioRxiv preprint doi: https://doi.org/10.64898/2026.01.28.702318; this version posted February 1, 2026. The copyright holder for this preprint (which was not certified by peer review) is the author/funder, who has granted bioRxiv a license to display the preprint in perpetuity. It is made available under aCC-BY 4.0 International license.

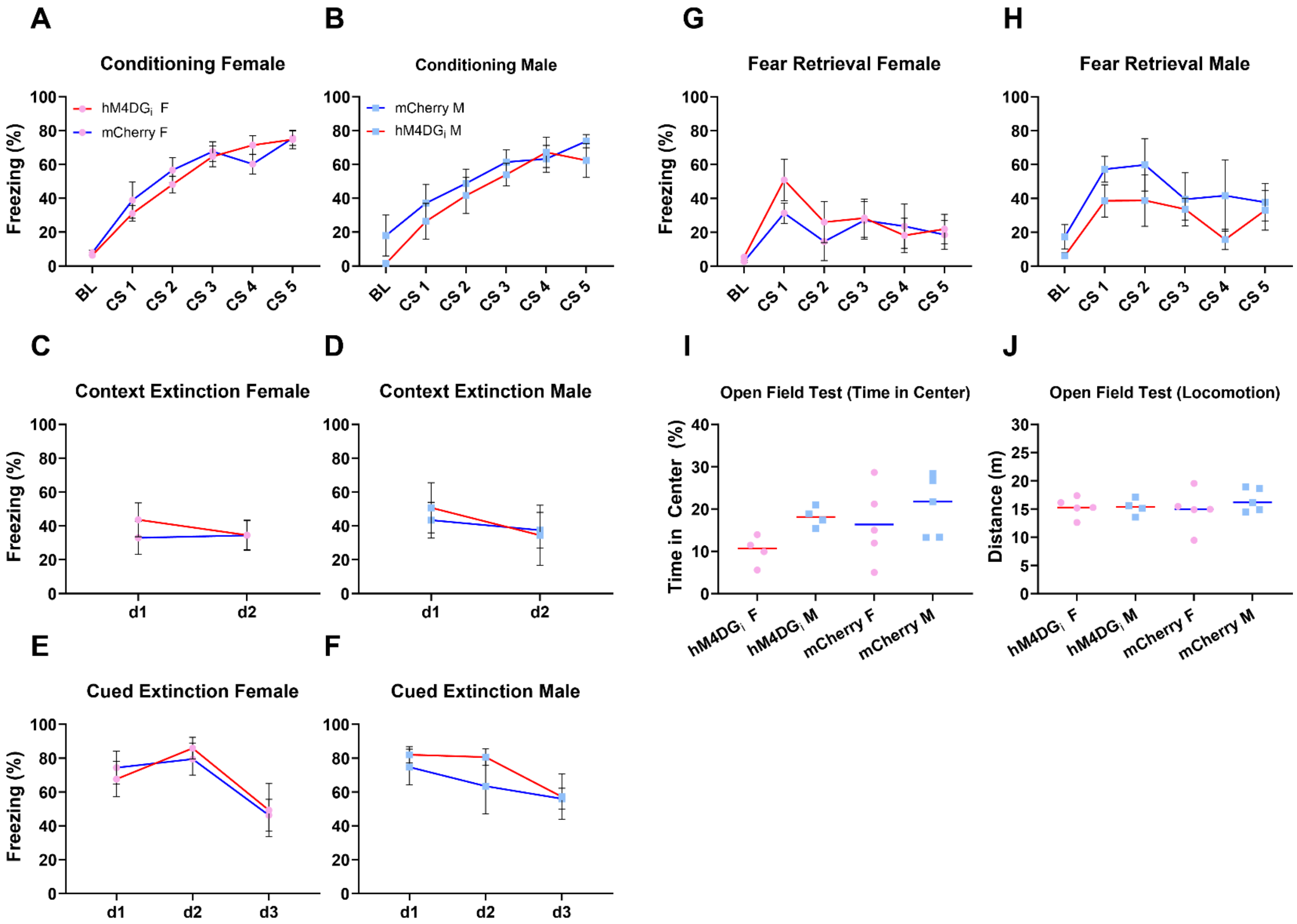

Fig. S2. DREADD inhibition of Cst+ neurons during cued extinction has no effect on extinction learning. A-B) Both males and females condition normally to footshock-tone presentations. C-H) No differences are observed in contextual fear extinction (C-D), cued fear extinction (E-F), or extinction retrieval (G-H). (I-J) No significant differences were observed in open field testing in time spent in center (I) or total locomotion (J)
